# The role of the clinical pharmacist in antimicrobial stewardship in Asia: A review

**DOI:** 10.1017/ash.2022.310

**Published:** 2022-11-07

**Authors:** Kittiya Jantarathaneewat, Bernard Camins, Anucha Apisarnthanarak

**Affiliations:** 1 Center of Excellence in Pharmacy Practice and Management Research, Faculty of Pharmacy, Thammasat University, Pathum Thani, Thailand; 2 Research Group in Infectious Diseases Epidemiology and Prevention, Faculty of Medicine, Thammasat University, Pathum Thani, Thailand; 3 Division of Infection Diseases, Department of Medicine, Icahn School of Medicine at Mount Sinai, New York, New York, United States; 4 Division of Infectious Diseases, Department of Internal Medicine, Faculty of Medicine, Thammasat University, Pathum Thani, Thailand

## Abstract

Clinical pharmacist-driven antimicrobial stewardship programs (ASPs) have been successfully implemented. Although relevant guidance and several studies suggest that clinical pharmacists be integrated into the current ASP team model, barriers still exist in Asia, primarily due to lack of dedicated personnel and lack of career advancement. We review the effectiveness and the ideal role of clinical pharmacist among ASPs in Asia. Several studies conducted in Asia have shown the effectiveness of pharmacist-led ASP interventions in hospitals and other healthcare settings. However, opportunities to expand the role of clinical pharmacists in ASPs in Asia exist in the implementation of rapid diagnostic test and drug allergies.

Infections caused by multidrug-resistant (MDR) pathogens can lead to high morbidity and mortality among hospitalized patients. In 2019, the global mortality associated with MDR pathogens was estimated to be ∼4.95 million deaths and the most common drug-resistant pathogens that led to death were *Escherichia coli*, *Staphylococcus aureus*, *Klebsiella pneumoniae*, *Streptococcus pneumoniae*, *Acinetobacter baumannii*, and *Pseudomonas aeruginosa*.^1^ Carbapenem-resistant gram-negative bacteria, such as *A. baumannii* and Enterobacterales, are of grave concern in the United States as well as the European continent and the Asia-Pacific region.^2–4^ In the era of MDR pathogens, the availability of active antimicrobial agents has become limited. Therefore, to combat these MDR pathogens, several strategies must be incorporated such as improved infection prevention and control, implementation of antimicrobial stewardship (ASP), active surveillance, and development of new antimicrobial agents. In well-developed countries like the United States, it is well recognized that the ASP team should consist of multidisciplinary healthcare personnel (eg, infectious diseases physician, clinical pharmacist, infection control nurse, and clinical microbiologist) who can influence appropriate antimicrobial prescriptions. Integration of the pharmacist into the ASP team has been shown to increase appropriateness of antimicrobial prescriptions and reduction in antimicrobial consumption, hospital antimicrobial expenditure, and in hospital length of stay.^5–7^

Guidelines from the Infectious Diseases Society of America (IDSA), the American Society of Health-System Pharmacist (ASHP), and a consensus statement on ASP from Asia recommended that a pharmacist should be a coleader of the ASP.^8–10^ The clinical pharmacist member of the ASP team should have some training in infectious disease. Clinical pharmacists are core members of the ASP team and have an important role in ensuring appropriate antimicrobial prescriptions. According to an ASHP statement, a pharmacist involved in the ASP has responsibilities to promote optimal antimicrobial use, to reduce the transmission of infections, and to educated other healthcare professionals, patients, and the public.^8^ The role of the ASP clinical pharmacist includes developing local guidance and formulary choices with the physician coleader, recommending switching from intravenous to oral formulations, identifying de-escalation opportunities, optimizing antimicrobial dose, monitoring drug–drug and drug–food interactions, and monitoring the outcomes of antimicrobial usage (eg, clinical outcomes and antimicrobial consumption). In addition, clinical pharmacists can help educate healthcare personnel on the appropriate use of antimicrobials as part of the processes of patient care.^11^ A study in the United States showed that an infectious-diseases–trained pharmacist on the ASP team is associated with reduced antimicrobial consumption, reduced mortality associated with sepsis and respiratory tract infections, and reduced antimicrobial costs.^12^ However, barriers to the integration of the clinical pharmacist into the ASP team in general, include the lack of financial support for dedicated personnel, lack of time, lack of career promotion for the pharmacist, and lack of knowledge and experience. Therefore, continued recommendations for clinical pharmacist engagement in the ASP team is essential. We reviewed the literature on the role and effectiveness of the clinical pharmacist in ASP in Asia, with a primary focus on the adult inpatient setting. The relevant studies were identified by searching the PubMed, ScienceDirect, and EMBASE databases from their inception date through August 2022. In this review, included studies were limited to English-language publications. The search strings “pharmacist,” “asia OR bangladesh OR brunei OR bhutan OR darussalam OR cambodia OR china OR guam OR hong kong OR india OR indonesia OR japan OR korea OR lao people’s democratic republic OR macao OR malaysia OR mongolia OR myanmar OR nepal OR northern mariana islands OR palau OR papua new guinea OR philippines OR singapore OR taiwan OR thailand OR timor-leste OR vietnam,” and “antimicrobial stewardship OR antibiotic stewardship” were used to identify papers that met the inclusion criteria. Our outcomes of interest were clinical outcome, proportion of appropriate antibiotic prescription, antibiotic consumption, antibiotic resistance rates, and pharmacist’s rate of recommendation acceptance. We only included papers that mainly emphasized pharmacist-led antimicrobial stewardship interventions. Letters to the editor, editorials, commentaries, review articles, and conference abstracts were excluded. The title and abstracts were screened for eligibility, and data extraction was conduted by K.J. and A.A.

## Role of the clinical pharmacist in the ASP team

Several studies from around the world have reported that clinical-pharmacist–led interventions have been successfully implemented. These interventions included performing a prospective audit and feedback, educating healthcare professionals, developing guidance for treatment a specific infection (eg, urinary tract infection and respiratory tract infection) and a specific pathogen (eg, *Staphylococcus aureus* and *Clostridioides difficile*), encouraging penicillin allergy delabeling, and facilitating real-time pathogen identification and feedback to the treating physician.^13–20^ The infectious diseases (ID) pharmacist is the ideal role model for pharmacist-led ASP interventions. Various studies have supported the effectiveness of ID pharmacist-led ASP interventions.^12,13^ However, most successful clinical-pharmacist–led interventions have been performed in high-income countries such as the United States, Canada, Australia, and Japan, as well as the European continent.

## Evidence on the efficacy of pharmacist-driven ASPs in Asia

**Fig. 1.**
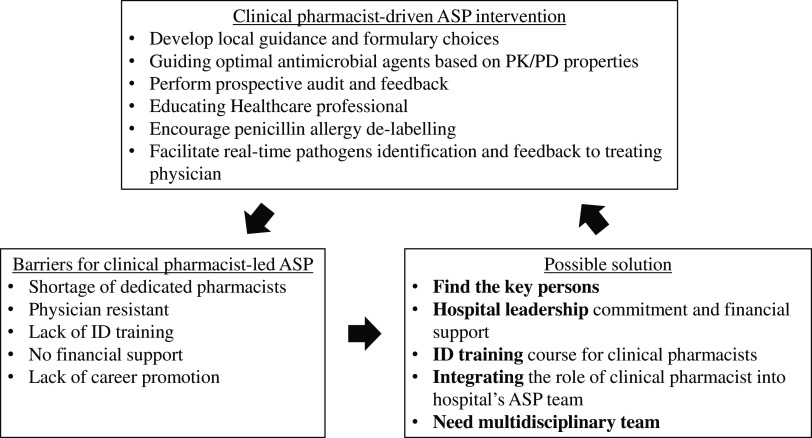
Barriers and solutions for clinical pharmacist-driven ASP intervention in Asia.

Several studies have reported the effectiveness of ID-pharmacist–driven ASPs in Asian countries such as Japan, Thailand, China, Korea, and India (Table 1). In Japan, ID pharmacist involvement in the ASP of a tertiary-care hospital increased the rate of appropriate blood-culture collection and de-escalation therapy (71% vs 85%; *P* < .001).^21^ A community hospital ASP with clinical pharmacist involvement decreased the duration of antimicrobial treatment in uncomplicated gram-negative bacteremia (8 vs 14 days; *P* < .001) and resulted in an increase in de-escalation, as reported in the study by Nakamura et al (10.2% vs 30.8%; *P* < .05).^22,23^ A clinical-pharmacist–led ASP intervention among patients with methicillin-resistant *Staphylococcus aureus* (MRSA) bacteremia led to an increase in targeted antimicrobial administration duration (at least 14 days for uncomplicated bacteremia and 28 days for complicated bacteremia; 44.8% vs 72.1%; *P* = .027) and early use of anti-MRSA drugs within 24 hours after MRSA was detected (62.3% vs 82.4%; *P* =.038).^24^ A timeout intervention for vancomycin showed a decrease in weekly vancomycin days of therapy (DOT) per 1,000 patient day (coefficient, −0.49; *P* = .007) as well as a decrease antimicrobial usage in the pharmacist-led arm (coefficient, −0.77; *P* = .007) compared to an ID physician arm.^25^ Finally, a study in Japan reported that clinical pharmacist involvement in an ASP team reduced the volume of antimicrobial prescriptions in a skilled nursing facility (incidence rate ratio [IRR], 0.885; *P* < .001) (Figure 1).^26^

**Table 1.** Summary of studies on ASP with clinical pharmacist involvement in Asia classified by countries

Country	Type of study and population	Intervention	Primary outcomes	Secondary outcomes
Japan				
Uda A, et al. 2022^21^	Study design: Pre and post intervention Study period: 2017-2019 Setting: Tertiary-care hospital Participants: N/A	ID Pharmacist perform a daily structured review of antibiotic prescriptions, Educating prescribers on antimicrobial therapy, Monthly reporting of department-level rates of blood sampling for culture Comparator: baseline period (May-Dec 2017) vs Intervention period (May-Dec 2018) and post-intervention period (May-Dec 2019	- Increased rate of appropriate blood culture collections and de-escalation therapy (71% vs 85%, *P* < .001)	- Decrease in antipseudomonal agent and carbapenem consumption (*P* = .016 and *P* = .004) - Decrease incidence of HA-CDI (*P* = .031) - Decrease 30-day mortality (*P* = .005) - Similar length of stay
Fukuda T, et al. 2021^22^	Study design: Retrospective cohort Study period: 2013-2015 Setting: uncomplicated gram-negative bacteremia patient, community hospital Participants: 66	Pharmacists perform an antimicrobial time-out at 72 hours after blood culture collection, optimized treatment based on the patient’s clinical response and test results Comparator: no pharmacist and no ID physician	- Decrease duration of antimicrobial treatment (8 vs 14 days, *P* < .001)	- Higher trend of de-escalation rate (P = .08) - Similar clinical success and failure, recurrence, infectious diseases re-admission, *Clostridioides difficile* infection, and 30- and 60-day mortality rates
Nakamura S, et al. 2021^23^	Study design: Retrospective study Study period: 2018-2019 Setting: Community hospital Participants: 535	Pharmacists perform daily follow up, ASP team weekly round Comparator: preintervention period	- Increase in rate of orders for blood culture (56.3% vs 73.3%, *P* < .01) - Increased rate of de-escalation (10.2% vs 30.8%, *P* < .05) - Decrease in piperacillin/tazobactam and carbapenems consumption (*P* < .01)	- No different in 30-day mortality - Decrease 30-day recurrence rate (14.7% vs 7.5%, *P* < .05) - Acceptance rate of pharmacist’s intervention 94.1%
Ohashi K, et al. 2018^24^	Study design: Historical-control trial Study period: N/A Setting: MRSA bacteremia patient, Municipal hospital Participants: 94	Pharmacists received an alert of blood cultures positive for MRSA and immediate intervention according to the bundle Comparator: preintervention period	- Increase in compliance rate with the appropriate duration of therapy (44.8% vs 72.1%, *P* = .027), early use of anti-MRSA drugs (62.3% vs 82.4%, *P* = .038), higher rate of negative follow-up blood cultures (40% vs 80%, *P* < .001)	- Decrease in 30-day mortality (41.8% vs 21.6%, *P* = .044) - Decrease in hospital mortality (58.1% vs 27.5%, *P* = .003)
Hasegawa S, et al. 2021^25^	Study design: Pre and post intervention, crossover trial Study period: 2018-2019 Setting: inpatient tertiary care hospital Participants: 587 patients prescribed IV vancomycin	Time-out intervention between clinical pharmacist-led time-out arm and an ID physician-led time-out arm Comparator: pre-prescription authorization	- Decrease in weekly vancomycin DOT per 1000 patient-day in phase 2 (coefficient −0.49, *P* = .007) - Decrease in antimicrobial usage in the pharmacist-led arm (coefficient −0.77, *P* = .007)	- Higher proportion of vancomycin discontinuations within 72 hours in phase 2 - Similar mean vancomycin use, median length of stay, and in-hospital mortality
Takito S, et al. 2020^26^	Study design: pre and post intervention Study period: 2013-2017 Setting: Skilled nursing facility Participants: N/A	Pharmacists provided prescription recommendations to physicians based on Gram stain results Comparator: preintervention period	- Reduction in the slope of total number of all antimicrobials prescriptions per 100 residents per month (IRR 0.885, *P* < .001)	- Decrease in number of prescriptions for macrolides and fluoroquinolones per 100 residents per month (*P* = .154 and .753) - Similar number of prescriptions for cephalosporins per 100 residents per month
Thailand				
Apisarnthanarak A, et al. 2015^5^	Study design: Quasi-experimental, prospective, concurrent groups Study period: Jan 2012-Sep 2012 Setting: Medicine ward, tertiary-care hospital Participants: 574	Pharmacists perform PPAF Comparator: standard of care	- Less likely to use antibiotic inappropriately (*P* < .001)	- More antibiotic de-escalation (*P* < .001) - less duration of antibiotic use < 7 days (*P* < .001) - Shorter hospital length of stay (*P* < .001) - No difference in mortality
Rattanaumpawan P, et al. 2018^6^	Study design: Randomized controlled trial Study period: Feb – Sep 2013 Setting: Medicine ward, tertiary-care hospital Participants: 1632	Pharmacists perform PPAF Comparator: ID clinicals fellow perform PPAF	- Non-inferiority in clinical response rate (44.9% vs 39.7%, *P* = .20, difference 5.15%, 95% CI 2.69 – 12.98%)	- Similar in microbiological outcomes, antibiotic-associated complications, antibiotic consumption, antibiotic expenditure, and length of stay
Jantarathaneewat K, et al. 2021^27^	Study design: Quasi-experimental, prospective, concurrent groups Study period: Aug 2019 – April 2020 Setting: Febrile neutropenic patient, tertiary-care hospital Participants: 90	Pharmacist performed daily PPAF and education Comparator: standard ASP	- Increase appropriateness of prescription (88.9% vs 51.1%, *P* < .001)	- Trend to lower meropenem, ceftazidime and cefepime similar 30-day ID mortality and length of stay - Acceptance rate of pharmacist’s recommendation 93.8%
Jantarathaneewat K, et al. 2022^13^	Study design: Quasi-experimental, prospective, concurrent groups Study period: 2019-2020 Setting: Medicine ward, tertiary-care hospital Participants: 400	ID pharmacist Daily prospective audit and feedback Comparator: standard ASP group	- Guideline adherence was higher in intervention group (79% vs 56.6%, *P* < .001)	- Trend to decrease 30-day all-cause mortality (15.9% vs 1.5%, *P* = .344) - Trend towards improved Clinical cure rate (63.6% vs 56.1%, *P* = .127) - Decrease Carbapenems consumption (*P* =.042) - Decrease Incidence of multidrug resistant pathogens (*P* = .049) - Similar Length of stay (*P* = .085)
China				
Li Z, et al. 2017^30^	Study design: Prospective cohort study Study period: March – April 2014 Setting: Intensive care units, multicenter Participants: 577	Pharmacists in 4 ICUs perform daily ward round and communicate with physician when inappropriate are prescribed Control: other 4 ICUs without pharmacists’ involvement	- Lower all-cause hospital mortality (19.3% vs 29.0%; *P* = .007)	- Reduce multidrug resistance (31.7% vs 23.8%, *P* = .037) - Less inappropriate third- and fourth-generation cephalosporin initiation (9.1% vs 15.2%, *P* = .031) - Shorter duration of mechanical ventilation (*P* = .184), length of stay in ICU (*P* = .227), and length of stay in hospital (*P* = .544) - Acceptance rate of pharmacist’s recommendation 71.9%
Du Y, et al. 2020^31^	Study design: Retrospective study Study period: 2016-2018 Setting: Gastroenterology ward, tertiary-care hospital Participants: 1763	Pharmacists perform daily ward rounds with physicians, regular review of medical orders, monthly indicator feedback, frequent physician training, and necessary patient education Comparator: pre intervention period	- Decrease in intensity of antibiotic consumption (coefficient −0.88, *P* = .01)	- Decrease in proportion of patients receiving combined antibiotics (coefficient −9.91, *P* = .03) - Decrease in average length of hospital stay (coefficient −1.79, *P* = .00) - Temporary increase in patients receiving antibiotics (coefficient 4.95, *P* = .038)
Zhou Y, et al. 2015^32^	Study design: prospective study with historical controls Study period: 2010-2013 Setting: Department of urology, tertiary-care hospital Participants: 234	Pharmacists led the antibiotic stewardship program at the hospital and on the urological clinical service; performed prospective audits and feedback from 2011 to 2013 Comparator: no pharmacist involvement period (2010)	- Decrease in antibiotic use density by 58.8% - Average antibiotic cost decreased by US $246.94	N/A
Xu J, et al. 2022^33^	Study design: Quasi-experimental, concurrent groups, retrospective Study period: 2018-2019 Setting: Department of Vascular and interventional radiology, Tertiary hospital Participants: 1026	Pharmacists perform Daily PPAF and guideline development Comparator: ASP team without pharmacist group	- Average score of inappropriate antimicrobials decreases in intervention group: perioperative antimicrobial prophylaxis (coefficient −0.207, *P* < .001), Non-surgical antimicrobial prophylaxis (coefficient −0.164, *P* = .010), Therapeutic use of antibiotics (coefficient −0.0694, *P* = .003)	- Decreased antimicrobial consumption (*P* = .017) - Decreased antimicrobial cost (*P* = .006) - Decreased average cost per defined daily dose (*P* < .001) - Similar total cost of hospitalization (*P* = .476) and length of hospital stay (*P* = .375) - Acceptance rate of pharmacist’s recommendation 52.78% for non-surgical prophylaxis, 76.69% for surgical prophylaxis, 86.18% for treatment
Zhou H, et al. 2021^34^	Study design: pre-and postintervention study Study period: June 2018- March 2019 Setting: Orthopedics department in a tertiary-care hospital Participants: 873	Pharmacist-led ASP team participated in ward rounds, reconciled patient’s allergy history, education, and performed standard intradermal skin test with the perioperative antibiotic prophylaxis regimen Comparator: pre-intervention period	- Decrease in the utilization of intradermal skin tests, from 95.8% to 16.5% (*P* < .001) - More cephalosporins used as prophylactic antimicrobial (*P* < .001) - Reduced antimicrobial expenditure by US $150.21 (*P* < .001) for each patient	- postintervention population was less likely to undergo an intradermal skin test (OR: 0.008, 95% CI: 0.005–0.014) - Patients in postintervention group had a 5.3-fold higher likelihood (95% CI: 2.95–9.43) of having cephalosporin as prophylactic antimicrobials
Wang H, et al. 2019^35^	Study design: Retrospective study Study period: July 2010- Dec 2016 Setting: Tertiary-care hospital Participants: patients with outpatient prescription (17,766,637) and inpatient prescriptions (376,627)	ASP interventions led by pharmacists such as formulating the activity program and performance management, advising on antibacterial prescriptions and training Comparator: baseline period	- Decreased in the number of antibiotic prescriptions in the inpatient setting by 59% (*P* < .05) and the outpatient setting by 33% (*P* < .05) each month - Decreased number of of antibiotic prophylaxis by 5.71% (*P* < .001) each month - Decrease DDD from 102.46 to 37.38 DDD/100 bed-days (*P* < .05) - Significant decrease in resistance rates among *E. coli* and *P. aeruginosa* isolates to fluoroquinolones - Significant decrease in the incidence of MRSA - Significant increase in resistance rates of *E. coli* and *K. pneumoniae* to carbapenems	- Increase rational timing of initial dose and rational duration
Zhang J, et al. 2020^36^	Study design: prospective, multicenter cohort study Study period: April 2017- Dec 2019 Setting: 17 acute care hospitals across Guizhou Province Participants: 2663 with confirmed infections	Pharmacist conducted a chart review and provided recommendation to clinician	- More effective clinical response was observed in patients whose provider accepted the ASP recommendation intervention group (81.34% vs 67.16%, *P* < .001)	- non-ID pharmacist showed similar effective clinical response to ID pharmacist (*P* = .896) - Acceptance rate of pharmacists ‘recommendation was 5.0%
Xu S, et al. 2021^37^	Study design: retrospective, observational pre-and post-intervention study Study period: 2018-2020 Setting: tertiary-care hospital Participants: 524 patients with community-acquired pneumonia eligible for IV to PO conversion	Prescribers were contacted by pharmacists about patients who were eligible for IV to oral antibiotic switches through computer- generated messages (phase 2) Comparator: prescribers were contacted bypharmacists who verbally informed them of patients who were eligible for an IV to oral conversions (phase 1)	- Increased proportion of patients who were converted to oral therapy on the day they were eligible from 34.8% in phase 1 to 62.7% in phase 2 (*P* < .05)	- Shorter lengths of IV antibiotic therapy days and hospital stay (*P* < .05) - Similar total length of antibiotic therapy day (*P* > .05)
Korea				
Song JY, et al. 2015^38^	Study design: pre and post intervention Study period: 2013 Setting: patient who received double anti-aerobic activity, Tertiary-care hospital Participants: 313	Pharmacists perform education and PPAF along with ID physician Comparator: preintervention period	- Decrease number of patients receiving unnecessary double anti-aerobic activity more than 3 days (26.8 vs 7, *P* = .005) - Decrease proportion of patients receiving unnecessary double anti-aerobic activity more than 3 days (42.3% vs 13.6%, *P* < .001)	- Acceptance rate of pharmacist’s recommendation 93.9%
Suh Y, et al. 2021^39^	Study design: retrospective study Study period: Jan-March 2017 Setting: multicenter, Tertiary hospital Participants: 4995	ASP with pharmacist involvement who intervene in antimicrobial prescription, perform TDM and monitored antimicrobial-related adverse drug event Comparator: ASP without pharmacist involvement	- Less incidence proportion of antimicrobial-related adverse event (8.9% vs 14.7%, *P* < .001)	- multidisciplinary ASPs including clinical pharmacists reduced the risk of antimicrobial-related ADEs by 38% (adjusted odds ratio 0.62; 95% CI 0.50–0.77)
India				
Nampoothiri V, et al. 2021^40^	Study design: Descriptive study Study period: 2016-2017 Setting: Tertiary care hospital Participants: 1326	Pharmacists perform daily PPAF Comparator: baseline period	- Increase appropriateness to 80% in the third year of intervention period - Decrease in antimicrobial consumption	- Decrease in antimicrobial consumption - Acceptance rate of pharmacist’s recommendation 70%

DDD, defined daily dose; HA-CDI, hospital-acquired *Clostridioides difficile* infection; ID, infectious diseases; IRR, incidence rate ratios; IV-PO conversion, intravenous to oral antibiotic conversion ; MRSA, Methicillin-resistant *Staphylococcus aureus*; TDM, therapeutic drug monitoring; PPAF, prospective audits and feedback.

Several clinical-pharmacist–led ASP studies have been conducted in Thailand. A randomized controlled trial in Thailand found that an ASP clinical-pharmacist–led prospective audit and feedback was not inferior to that of an ID fellow in terms of clinical response (difference, 5.15%; 95% CI, 2.69%–12.98%). This finding confirms the concept that a clinical pharmacist can be an alternative to a physician-led ASP implementation strategy.^6^ This research was followed by a study conducted in a medicine ward at a tertiary-care hospital that showed that an ID-pharmacist–led ASP intervention, especially in conjunction with an ID physician, can further increase guideline adherence (79% vs 56.6%; *P* < .001), as well as decrease carbapenem consumption (mean defined daily dose [DDD], 191.94 vs 256.14; *P* = .042).^5,13^ In the specific setting of febrile neutropenic patients, a clinical-pharmacist–led ASP intervention was associated with more appropriate prescriptions (88.9% vs 51.1%; *P* < .001) without affecting the mortality rate.^27^ Finally, clinical pharmacist involvement in the multidisciplinary team support improved adherence to a vancomycin dosing protocol (90.8% vs 55%; *P* < .001), and the clinical pharmacist can help the ASP team implement antibiotic heterogeneity through periodic antibiotic monitoring and supervision strategy in both medicine and surgical departments in Japan and Thailand.^28,29^

In China, a multicenter study conducted in 8 intensive care units (ICUs) at 4 university hospitals resulted in a lower all-cause hospital mortality in 4 ICUs where clinical pharmacists were involved (19.3% vs 29%; *P* = .007).^30^ But this study had limitations including a short study period, potential selection bias of the clinical cohort, and a small sample size.^30^ Studies conducted in a gastroenterology ward and a urology ward, respectively, reported reductions in antimicrobial consumption during the respective study periods in which clinical pharmacists were involved (coefficient, −0.88; *P* = .01).^31,32^ A quasi-experimental study on the efficacy of an ID pharmacist with a concurrent control group in the department of vascular and interventional radiology reported a decrease in inappropriate prescriptions, particularly in perioperative antimicrobial prophylaxis (coefficient, −0.207; *P* < .001), nonsurgical antimicrobial prophylaxis (coefficient, −0.164; *P* = .010), and therapeutic use of antimicrobials (coefficient, −0.069; *P* = .003).^33^ In an orthopedic department, a pharmacist-led intervention was performed on perioperative antibiotic prophylaxis by standardizing the cephalosporin intradermal skin test.^34^ This intervention achieved a reduction the utilization of intradermal skin tests from 95.8% to 16.5% (*P* < .001) and reduced the cost of antimicrobials by $150.21 (*P* < .001) for each patient.^34^ A pharmacist-led ASP, hospital-wide intervention in China improved antibiotic consumption and achieved a corresponding decrease in the incidence of drug-resistant gram-negative bacteria.^35^ A multicenter study also showed that adherence to clinical pharmacist’s recommendations improved clinical response compared with rejection of the clinical pharmacist’s recommendation.^36^ Among patients with community-acquired pneumonia, a pharmacist-led intervention converting intravenous to oral administration of antibiotic integrated with computer decision support led to an increase in the proportion of patients who were converted to oral therapy from 34.8% to 62.7% (*P* < .05) and shortened the lengths of intravenous antibiotic therapy and hospital stay (*P* < .05).^37^

In South Korea, a clinical pharmacist-led intervention that focused on patients receiving antimicrobials with redundant activity.^38^ The ID-pharmacist–led intervention achieved a decrease in the number (26.8 vs 7; *P* = .005) and proportion (42.3% vs 13.6%; *P* < .001) of patients receiving unnecessary antimicrobials for >3 days.^38^ Another retrospective study in South Korea reported that an ASP with clinical pharmacist involvement was associated with a decrease in the incidence of antimicrobial-related adverse events (8.9% vs 14.7%; *P* < .001).^39^ Finally, similar to previous studies, a study on clinical pharmacist involvement in the ASP in India showed an increase in the proportion of appropriate prescriptions during the third year of the intervention period.^40^

Similar to those reported in Western developed countries like the United States, the acceptance rates of clinical pharmacist recommendations in Asia range from 70% to 94%. In high-income Asian countries, such as Japan and South Korea, the acceptance rates of pharmacist recommendations tend to be higher (∼90%), whereas in middle-income countries, such as China and India, tend to report lower acceptance rates (70%–90%). The most effective interventions feature daily prospective audit and feedback by the clinical pharmacist along with the ID physician.^41^ The relevant outcomes in Asia include adherence to the intervention or bundle, appropriateness of antimicrobial use, antimicrobial consumption, and clinical outcomes such as clinical improvement and mortality. An ASP that includes clinical pharmacists can increase the appropriateness of antimicrobials prescribed and decrease unnecessary antimicrobial use. However, rare reports of the impact of intervention on the development of antimicrobial resistance have emerged, and they may indicate the need to integrate effective infection prevention. The other relevant outcomes, such as cost-effectiveness of clinical pharmacist involvement in the ASP team, should be the subject of future research. Clinical-pharmacist–led ASP interventions in other settings such as ambulatory care, emergency room, high dependency unit, pediatrics, solid-organ transplant unit, patients with cytomegalovirus infection, and patients with invasive candidiasis have been conducted in the United States, Canada, and Europe.^42–48^ The addition of delabeling penicillin allergies through penicillin skin testing by the clinical pharmacist to current ASP interventions has effectively reduced antimicrobial-therapy–related costs, clarification of allergies, and optimized antimicrobial therapy.^49,50^ Additionally, during the COVID-19 pandemic, ASP interventions were essential to optimizing antimicrobial use when rates of bacterial coinfection were relatively low.^51^ In Canada, a clinical trial of a clinical pharmacist-led ASP team on COVID-19 patients is ongoing.^52^ Interventions on COVID-19 patients have not been well recognized in Asia; thus, longer follow-up periods with larger sample sizes are needed.

A study in the United States showed that the use of procalcitonin levels to guide interventions by clinical pharmacists led to discontinuation of vancomycin therapy in patients with lower respiratory tract infections and normal procalcitonin levels.^53^ Rapid identification of gram-positive bacteria through diagnostic systems like the VITEK-2 automated system, matrix-assisted laser desorption ionization–time-of-flight mass spectrometry (MALDI-TOF MS), and rapid polymerase chain reaction (PCR) with real-time ASP interventions by a clinical pharmacist have been implemented successfully in the United States.^19,20,54,55^ Identification of antimicrobial susceptibility using Verigene along with ASP guidance can improve time to initiation of appropriate antimicrobials as well as reduce mortality rates and 30-day readmission rates.^55^ Therefore, clinical pharmacist-led ASP interventions should be integrated with rapid diagnostic test.

## Barriers and possible solutions

In Asia, some barriers to the implementation of the ID pharmacist-led ASP remain. These include the lack of dedicated personnel, a labor-intensive pharmacist workload, lack of time, lack of knowledge and perception toward ASP by clinicians, shortage of clinical pharmacists with advanced training in infectious diseases, and the lack of integration of the pharmacists’ role with other healthcare professionals when delivering care.^13^ A survey in Malaysia revealed that administration support, commitment from leadership, and perseverance are essential to facilitate the role of clinical pharmacists in the ASP.^56^ Another survey in South Korea revealed that ASPs were limited only to top-tier general hospitals. The study concluded that a clinical pharmacist is essential to successful implementation of the ASP along with adequate financial reimbursement for the professionals who perform and maintain the ASP.^57^ Data from the survey of gaps and opportunities in ASPs in Asia revealed that 80% of respondents had a pharmacist working on ASP activities; however, almost half of the respondents reported no financial support such as salary support, training, or information technology services at their hospitals.^58^ Furthermore, a survey conducted in the Asia-Pacific region revealed that 41% of hospitals had no trained ID pharmacists even though most respondents worked in large hospitals.^59^ A nationwide survey from the United States revealed that pharmacists with formal ASP responsibilities dedicated 0.6 full-time equivalents (FTE), whereas pharmacists without formal ASP responsibilities spent an average of 0.125 FTE on ASP activities, even though the average program should have 1 FTE allocated for an ASP pharmacist.^60^ The lack of data on clinical-pharmacist FTE among ASPs in Asia may be a barrier to implementing effective ASPs in this region.

To successfully encourage pharmacist involvement in their respective ASPs, each institution should identify its own gaps and challenges.^9^ Prioritizing formal support and approval from hospital leadership and making a case for the return on investment of clinical pharmacist involvement in ASP activities are essential.^9^ Illustrating to administrators that the role of the clinical pharmacist in the ASP team is that of an expert in antimicrobial delivery can potentially lead to a modification in their role in routine activities in the hospital pharmacy. A full-time antimicrobial stewardship pharmacist has been well recognized as a viable advanced career path for clinical pharmacists in the United States, Canada, and Europe.

In Asia, especially in developing countries, most ID-trained clinical pharmacists perform their ASP activities along with their routine work without additional renumeration. Without financial support from hospital administration, the ID-trained clinical pharmacist will not be able to complete their tasks on the ASP team. A possible solution is to require administrative support before hospitals are granted accreditation. The pharmacy council should promote the clinical pharmacist’s role in the ASP team and support the continuing education for pharmacist who are interested in or work for the ASP team. Furthermore, the role of the clinical pharmacist on the ASP team should be integrated into the curricula of doctor of pharmacy programs to encourage participation of pharmacists in the multidisciplinary ASP team. A recent study identified a trend toward increased clinical pharmacist involvement in ASP teams in Japan. These researchers also recommended that policy makers and stakeholders promote and support the evidence-based activities of clinical-pharmacist–led ASPs for small to medium-sized hospitals.^61^

This review had several limitations. It was difficult to determine which factors were important to the success of the ASP because each study involved a multifaceted intervention and different outcomes were measured. Our review is mostly applicable to the adult inpatient population rather than other settings such as the outpatient setting and pediatric population. Publication bias may exist, especially in the middle- and low-income countries. The standardized definitions for processes and outcome measures in ASP is necessary for future studies. Future studies that stratify interventions based on the income level of the country would provide more insight and would improve the homogeneity of the data. The role of clinical pharmacist in ASPs in Asia is limited to the inpatient setting. Further studies on other settings are needed before any recommendations can be made on pediatric and ambulatory settings.

According to previous studies, clinical pharmacist involvement in the ASP team can be implemented successfully in Asia. However, some barriers to widespread implementation of clinical-pharmacist–driven ASPs remain. The approval of and financial support from hospital leadership are important steps in initiating an ASP strategy. Integrating a clinical pharmacist’s role in an ASP team as a policy and financial support along with career advancement for the clinical pharmacist will encourage the proliferation and acceptance of clinical-pharmacist–driven ASPs. Training in infectious diseases is essential for clinical pharmacists on ASP teams. Moreover, the role of clinical pharmacist can be expanded if ASPs are implemented in other areas such as ambulatory care, the emergency departments, and units housing immunocompromised patients. Integrated rapid diagnostic methods and real-time ASP interventions are attractive strategies that will expand the clinical pharmacy service.
